# Supplementary material for: Continuous flow hydrogenation of methyl and ethyl levulinate: an alternative route to γ-valerolactone production
Source: R Soc Open Sci. 2019 May 1;6(5):182233. doi: 10.1098/rsos.182233 (PMC6549990; doi:10.1098/rsos.182233)
Supplement: Electronic Supplementary Material [file rsos182233supp1.docx]

Continuous flow hydrogenation of methyl- and ethyl-levulinate: an alternative route to γ-valerolactone production

József M. Tukacs,^a^ Áron Sylvester,^a^ Ildikó Kmecz,^a^ Richard V. Jones,^b^
Mihály Óvári^c^ and László T. Mika^a,^*

^a^Budapest University of Technology and Economics, Department of Chemical and Environmental Process Engineering, H-1111, Budapest, Műegyetem rkp. 3, Hungary, Tel: +361463 1263 E-mail: [laszlo.t.mika@mail.bme.hu](mailto:laszlo.t.mika@mail.bme.hu)

^b^ThalesNano Nanotechnology Inc., H-1031, Budapest, Záhony u. 7, Hungary

^c^MTA Centre for Ecological Research, Institute for Danube Research, H-1113, Budapest, Karolina u. 29, Hungary

**Electronic Supplementary Material**

Abbreviations: EL: ethyl levulinate, EHP: ethyl 4-hydroxypentanoate, GVL: gamma-valerolactone, ML: methyl levulinate, MHP: methyl 4-hydroxypentanoate, nd: not determined, X: Conversion (X = mol_converted subsrate_ × (mol_subsrate_)^–1^), P: Productivity P = mol_GVL_ × g_metal_^-1^ × h^-1^ , S: Selectivity (Selectivity = mol_product_ × (mol_product_ + mol_by-product_)^–1^), Y: yield (Y = mol_product_ × (mol_subsrate_)^–1^,

Fig. S1 Schematic representation of a continuous flow reactor: **H-Cube**^®^
(More information RSC. Adv. 2013, 3, 16283.)

Table S1. Effect of pressure on the synthesis of *γ*-valerolactone from ML using H-Cube^®^ as a continuous flow reactor system in 20 mL of water with a flow rate of 1 mL min^-1^ at 100 °C

| **#** | **p**  (bar) | **X_substrate_**  (%) | **Y_GVL_**  (%) | **Y_MHP_**  (%) | **S_GVL_**  (%) | **S_MHP_**  (%) | **P_GVL_**  (mol_GVL_×g_Metal_^-1^×h^-1^) |
| --- | --- | --- | --- | --- | --- | --- | --- |
| 1 | 50 | 74.5 | 9.2 | 65.3 | 12.3 | 87.7 | 0.079 |
| 2 | 70 | 88.9 | 18.4 | 70.5 | 20.7 | 79.3 | 0.157 |
| 3 | 100 | >99.9 | 52.6 | 47.4 | 52.6 | 47.4 | 0.451 |

Table S2. Effect of pressure on the synthesis of *γ*-valerolactone from EL using H-Cube^®^ as a continuous flow reactor system in 20 mL of water with a flow rate of 1 mL min^-1^ at 100 °C

| **#** | **p**  (bar) | **TPPTS** (mmol/L) | **X_substrate_**  (%) | **Y_GVL_**  (%) | **Y_EHP_**  (%) | **S_GVL_**  (%) | **S_EHP_**  (%) | **P_GVL_**  (mol_GVL_×g_Metal_^-1^×h^-1^) |
| --- | --- | --- | --- | --- | --- | --- | --- | --- |
| 1 | 50 | - | 47.5 | 17.0 | 30.5 | 35.7 | 64.3 | 0.146 |
| 2 | 70 | - | 80.8 | 30.7 | 50.1 | 38.0 | 62.0 | 0.263 |
| 3 | 100 | - | >99.9 | 50.6 | 49.3 | 50.6 | 49.3 | 0.434 |
| 4 | 50 | 1.5 | 65.5 | 21.7 | 43.7 | 33.2 | 66.5 | 0.187 |
| 5 | 70 | 1.5 | 78.2 | 25.3 | 52.9 | 32.3 | 67.7 | 0.217 |
| 6 | 50 | 15 | 90.0 | 58.1 | 31.9 | 64.5 | 35.5 | 0.498 |
| 7 | 70 | 15 | >99.9 | 66.1 | 33.9 | 66.1 | 33.9 | 0.567 |

Table S3. Effect of pressure on the synthesis of *γ*-valerolactone from ML using H-Cube^®^ as a continuous flow reactor system in 20 mL of methanol with a flow rate of 1 mL min^-1^ at 100 °C

| **#** | **p**  (bar) | **X_substrate_**  (%) | **Y_GVL_**  (%) | **Y_MHP_**  (%) | **S_GVL_**  (%) | **S_MHP_**  (%) | **P_GVL_**  (mol_GVL_×g_Metal_^-1^×h^-1^) |
| --- | --- | --- | --- | --- | --- | --- | --- |
| 1 | 50 | 74.6 | 20.6 | 54.0 | 27.6 | 72.4 | 0.177 |
| 2 | 70 | 81.1 | 23.4 | 57.7 | 28.8 | 71.2 | 0.201 |
| 3 | 100 | 93.7 | 29.6 | 64.1 | 31.6 | 68.4 | 0.254 |

Table S4. Effect of pressure on the synthesis of *γ*-valerolactone from EL using H-Cube^®^ as a continuous flow reactor system in 20 mL of ethanol with a flow rate of 1 mL min^-1^ at 100 °C

| **#** | **p**  (bar) | **X_substrate_**  (%) | **Y_GVL_**  (%) | **Y_EHP_**  (%) | **S_GVL_**  (%) | **S_EHP_**  (%) | **P_GVL_**  (mol_GVL_×g_Metal_^-1^×h^-1^) |
| --- | --- | --- | --- | --- | --- | --- | --- |
| 1 | 50 | 81.5 | 5.0 | 76.5 | 6.1 | 93.9 | 0.043 |
| 2 | 70 | 85.3 | 6.3 | 79.0 | 7.4 | 92.6 | 0.063 |
| 3 | 100 | 94.4 | 6.5 | 87.9 | 6.9 | 93.2 | 0.059 |

Table S5. Effect of the flow rate on the synthesis of *γ*-valerolactone from ML using H-Cube^®^ as a continuous flow reactor system in 20 mL of water under 100 bar of hydrogen at 100 °C

| **#** | **Flow rate**  (mL min^-1^) | **X_substrate_**  (%) | **Y_GVL_**  (%) | **Y_MHP_**  (%) | **S_GVL_**  (%) | **S_MHP_**  (%) | **P_GVL_**  (mol_GVL_×g_Metal_^-1^×h^-1^) |
| --- | --- | --- | --- | --- | --- | --- | --- |
| 1 | 0.2 | >99.9 | 76.8 | 23.2 | 76.8 | 23.2 | 0.131 |
| 2 | 0.5 | >99.9 | 62.2 | 37.7 | 62.2 | 37.7 | 0.266 |
| 3 | 0.7 | >99.9 | 58.8 | 41.2 | 58.8 | 41.2 | 0.352 |
| 4 | 1.0 | >99.9 | 49.9 | 50.1 | 49.9 | 50.1 | 0.428 |
| 5 | 1.5 | >99.9 | 47.6 | 52.4 | 47.6 | 52.4 | 0.612 |
| 6 | 2.0 | >99.9 | 44.1 | 55.9 | 44.1 | 55.9 | 0.755 |
| 7 | 2.5 | >99.9 | 42.5 | 57.5 | 42.5 | 57.5 | 0.910 |

Table S6. Effect of the flow rate on the synthesis of *γ*-valerolactone from EL using H-Cube^®^ as a continuous flow reactor system in 20 mL of water under 100 bar of hydrogen at 100 °C

| **#** | **Flow rate**  (mL min^-1^) | **X_substrate_**  (%) | **Y_GVL_**  (%) | **Y_EHP_**  (%) | **S_GVL_**  (%) | **S_EHP_**  (%) | **P_GVL_**  (mol_GVL_×g_Metal_^-1^×h^-1^) |
| --- | --- | --- | --- | --- | --- | --- | --- |
| 1 | 0.2 | >99.9 | 90.4 | 9.6 | 90.4 | 9.6 | 0.155 |
| 2 | 0.5 | >99.9 | 58.7 | 41.3 | 58.7 | 41.3 | 0.252 |
| 3 | 0.7 | >99.9 | 48.5 | 51.5 | 48.5 | 51.5 | 0.291 |
| 4 | 1.0 | >99.9 | 41.1 | 58.9 | 41.1 | 58.9 | 0.352 |
| 5 | 1.5 | >99.9 | 35.9 | 64.1 | 35.9 | 64.1 | 0.462 |
| 6 | 2.0 | >99.9 | 31.7 | 68.3 | 31.7 | 68.3 | 0.544 |
| 7 | 2.5 | >99.9 | 32.5 | 67.5 | 32.5 | 67.5 | 0.697 |

Table S7. Effect of the flow rate on the synthesis of *γ*-valerolactone from ML using H-Cube^®^ as a continuous flow reactor system in 20 mL of methanol under 100 bar of hydrogen at 100 °C

| **#** | **Flow rate**  (mL min^-1^) | **X_substrate_**  (%) | **Y_GVL_**  (%) | **Y_MHP_**  (%) | **S_GVL_**  (%) | **S_MHP_**  (%) | **P_GVL_**  (mol_GVL_×g_Metal_^-1^×h^-1^) |
| --- | --- | --- | --- | --- | --- | --- | --- |
| 1 | 0.2 | >99.9 | 22.9 | 77.0 | 22.9 | 77.0 | 0.039 |
| 2 | 0.5 | >99.9 | 16.5 | 83.5 | 16.5 | 83.5 | 0.071 |
| 3 | 0.7 | >99.9 | 13.5 | 86.5 | 13.5 | 86.5 | 0.081 |
| 4 | 1.0 | >99.9 | 12.2 | 87.8 | 12.2 | 87.8 | 0.104 |
| 5 | 1.5 | 82.6 | 10.7 | 71.8 | 13.0 | 87.0 | 0.138 |
| 6 | 2.0 | 64.5 | 9.0 | 55.5 | 13.9 | 86.1 | 0.146 |
| 7 | 2.5 | 25.7 | nd | 25.7 | nd | >99.9 | nd |

Table S8. Effect of the flow rate on the synthesis of *γ*-valerolactone from EL using H-Cube^®^ as a continuous flow reactor system in 20 mL of ethanol under 100 bar of hydrogen at 100 °C

| **#** | **Flow rate**  (mL min^-1^) | **X_substrate_**  (%) | **Y_GVL_**  (%) | **Y_EHP_**  (%) | **S_GVL_**  (%) | **S_EHP_**  (%) | **P_GVL_**  (mol_GVL_×g_Metal_^-1^×h^-1^) |
| --- | --- | --- | --- | --- | --- | --- | --- |
| 1 | 0.2 | >99.9 | 14.4 | 85.6 | 14.4 | 85.6 | 0.025 |
| 2 | 0.5 | >99.9 | 10.7 | 89.3 | 10.7 | 89.3 | 0.046 |
| 3 | 0.7 | >99.9 | 10.6 | 89.4 | 10.6 | 89.4 | 0.063 |
| 4 | 1.0 | >99.9 | 10.5 | 89.5 | 10.5 | 89.5 | 0.090 |
| 5 | 1.5 | 85.9 | 9.5 | 76.4 | 11.1 | 88.9 | 0.123 |
| 6 | 2.0 | 76.8 | 20.4 | 56.4 | 26.5 | 73.4 | 0.350 |
| 7 | 2.5 | 41.4 | nd | 41.4 | nd | >99.9 | nd |

Table S9. Investigation of long-term catalytic activity on the reduction of ML in water with a flow rate of 1 mL min^-1^ under 100 bar of hydrogen at 100 °C

| **#** | **Time**  (min) | **X_substrate_**  (%) | **S_GVL_**  (%) | **S_MHP_**  (%) | **[Ru]_leaching_**  (ppb) |
| --- | --- | --- | --- | --- | --- |
| 1 | 10 | >99.9 | 50.4 | 49.6 | 3.0 |
| 2 | 20 | >99.9 | 46.8 | 52.3 | 2.4 |
| 3 | 80 | >99.9 | 28.9 | 71.1 | <0.2 |
| 4 | 100 | >99.9 | 28.8 | 71.2 | <0.2 |
| 5 | 120 | >99.9 | 25.7 | 74.3 | <0.2 |
| 6 | 140 | >99.9 | 27.0 | 73.0 | <0.2 |
| 7 | 160 | >99.9 | 22.8 | 77.2 | <0.2 |
| 8 | 180 | >99.9 | 22.1 | 77.9 | <0.2 |
| 9 | 200 | >99.9 | 23.0 | 77.0 | <0.2 |
| 10 | 220 | >99.9 | 27.4 | 72.6 | <0.2 |
| 11 | 240 | >99.9 | 23.0 | 77.0 | <0.2 |

Table S10. Operating conditions of the ICP–SF–MS

| Plasma power | 1180 W |
| --- | --- |
| Outer gas (Ar) | 1.00 L/min |
| Intermediate gas (Ar) | 14.0 L/min |
| Aerosol carrier gas (Ar) | 0.83 L/min |
| Sample uptake | 0.30 mL/min |
| Nebulizer | Meinhard |
| Spray chamber | double pass |
| Sampler cone | Ni, 1.0 mm orifice |
| Skimmer cone | Ni, 0.7 mm orifice |
| Analytical isotope | ^101^Ru |
| Internal standard | ^115^In |
| Resolution of mass spectrometer | 4000 |
| Data acquisition | peak jumping |
| Dwell time | 0.1 s |
| Replicates | 15 |

The determination of all trace elements was carried out by external calibration against multi-elemental standard solutions; the relative error is about 1 to 2 %.
